# Isolation and Characterization of Buccal Fat Pad and Dental Pulp MSCs from the Same Donor

**DOI:** 10.3390/biomedicines9030265

**Published:** 2021-03-07

**Authors:** Tullio Genova, Davide Cavagnetto, Fabio Tasinato, Sara Petrillo, Federico Alessandro Ruffinatti, Luca Mela, Massimo Carossa, Luca Munaron, Ilaria Roato, Federico Mussano

**Affiliations:** 1 Department of Life Sciences and Systems Biology, University of Torino, Via Accademia Albertina 13, 10123 Torino, Italy; tullio.genova@unito.it (T.G.); federicoalessandro.ruffinatti@unito.it (F.A.R.); luca.munaron@unito.it (L.M.); 2 Department of Surgical Sciences, University of Torino, Via Nizza 230, 10126 Torino, Italy; tasinatofabio@gmail.com (F.T.); medmel@hotmail.com (L.M.); massimo.carossa@unito.it (M.C.); ilaria.roato@unito.it (I.R.); 3 Department of Molecular Biotechnology and Health Sciences, University of Torino, Via Nizza 52, 10126 Turin, Italy; sara.petrillo@unito.it

**Keywords:** mesenchymal stem cells (MSC), dental pulp stem cells (DPSCs), buccal fat pad stem cells (BFPSCs), oral cavity, regenerative medicine, tissue engineering

## Abstract

Mesenchymal stem cells (MSCs) can be harvested from different sites in the oral cavity, representing a reservoir of cells useful for regenerative purposes. As direct comparisons between at least two types of MSCs deriving from the same patient are surprisingly rare in scientific literature, we isolated and investigated the osteoinductive potential of dental pulp stem cells (DPSCs) and buccal fat pad stem cells (BFPSCs). MSCs were isolated from the third molar dental pulp and buccal fat pads of 12 patients. The number of viable cells was quantified through manual count. Proliferation and osteodifferentiation assays, flow cytometry analysis of cell phenotypes, and osteocalcin release in vitro were performed. The isolation of BFPSCs and DPSCs was successful in 7 out of 12 (58%) and 3 out of 12 (25%) of retrieved samples, respectively. The yield of cells expressing typical stem cell markers and the level of proliferation were higher in BFPSCs than in DPSCs. Both BFP-SCs and DPSCs differentiated into osteoblast-like cells and were able to release a mineralized matrix. The release of osteocalcin, albeit greater for BFPSCs, did not show any significant difference between BFPSCs and DPSCs. The yield of MSCs depends on their site of origin as well as on the protocol adopted for their isolation. Our data show that BFP is a valuable source for the derivation of MSCs that can be used for regenerative treatments.

## 1. Introduction

Human mesenchymal stem cells (MSCs) are the fundament of any tissue engineering approach aiming at regenerating mineralized tissues [1]. Historically, one of the first sites to derive MSCs has been bone marrow, but extraction is hindered by difficult accessibility and a frequently painful procedure [2]. Alternative sources of MSCs have been discovered, such as adipose tissue and dental pulp (DP), which can be easily and conspicuously harvested [3]. Adipose-derived stem cells (ASCs) are a plastic-adherent [4], possess a multipotent cell population attainable during liposuction, and rely on an impressive corpus of supporting research published since 2001 [5] reporting on their capability to differentiate into different tissues, such as bone and cartilage [6,7,8]. Likewise, ASCs can sustain the repair of large bone defects in vivo and have been tested in clinical studies with promising outcomes [9,10,11]. Extracted teeth have become an interesting source of MSCs since the first isolation of dental pulp stem cells (DPSCs) by Gronthos et al. in 2000 [3]. DPSCs have been proven able to promote bone regeneration at every level [1,12]. In vitro, DPSCs show high proliferation activity and are capable of osteogenic commitment [12,13]. These cells have consistently been reported to promote bone regeneration after in vivo transplantation [13]. Subsequently, stem cells were also isolated from the pulp of exfoliating deciduous teeth (SHED) [14,15], the periodontal ligament of permanent teeth (PDLSC) [16], and the apical papilla [17].

Recently, the buccal fat pad (BFP), usually called Bichat’s fat pad, emerged as a novel possible source of stem cells, known as buccal fat pad stem cells (BFPSCs). Indeed, studies reported on the successful repair of bone defects in the jaws using these cells as a pellet [18], or in combination with inorganic bovine bone mineral [19], enabling new protocols.

In this work, we exploit a particular surgical procedure in which maxillary wisdom teeth are extracted while avoiding possible oro-nasal communication. Thus, both the dental pulp and the buccal fat pad of the same patients were harvested, allowing us to isolate and characterize the DPSCs and BFPSCs. The fact that these two cell populations derived from the same patients allowed a direct comparison that is, to our knowledge, as unprecedented as it is useful. The objective of the present study was to assess which was the most promising source of mesenchymal stem cells between DPSCs and BFPSCs based on the yield of viable cells obtained and their osteodifferentiation potential. The null hypothesis was that there was no difference between the cell populations.

## 2. Materials and Methods

### 2.1. Patients and Ethics Issues

From January to May 2020, patients requiring the extraction of bone-included upper wisdom teeth and a subsequent flap to prevent oro-antral communication were referred to the Oral Surgery department of the Dental School of the University of Turin. Complete medical history, panoramic radiograms (OPTs), and cone–beam computed tomography of each patient were assessed. Cells from both the permanent teeth and the buccal fat pad were obtained from 12 clinically healthy patients (7 males and 5 females, mean age 24 ± 3 years, as reported in Table 1). As reported elsewhere [20], exclusion criteria were the following: “systemic or local disease or condition (hematologic diseases, uncontrolled diabetes, serious coagulopathies, history of intravenous therapy with bisphosphonates, and/or diseases of the immune system) possibly precluding oral surgical intervention; immunosuppression; HIV+, HCV+, HBV+, TBC+, corticosteroid treatment, pregnancy, radiotherapy to the head or neck region within 12 months before surgery.”

In this study, all clinical and laboratory procedures were approved by the Ethics Committee of the Hospital Città della Scienza e della Salute of Turin (approval number: 1753/2019, date 10/3/2019), in accordance with the 1964 Helsinki Declaration and its later amendments. Patients’ sensitive data were stored according to the EU law ensuring the full respect of privacy after receiving written informed consent.

### 2.2. Cell Isolation and Culture

#### 2.2.1. DPSCs

To prevent microbial contamination, teeth were rinsed quickly in a 5% sodium hypochlorite solution in water, then three times in Hank’s Balanced Salt Solution, and finally they were split using bone forceps. The DP tissue was minced and digested in a mixture of 3 mg/mL type I collagenase (Gibco/Invitrogen, Carlsbad, CA, USA), at 37 °C for 40 min. To discard extracellular debris, cells were filtered through a 70 μm cell strainer (Falcon; BD Labware, Franklin Lakes, NC, USA), then they were centrifuged and plated in a 35 mm dish containing alpha-modified Eagle’s medium (Gibco/Invitrogen, Carlsbad, CA, USA) supplemented with 10% fetal bovine serum (FBS; Sigma-Aldrich, Milan, Italy), 50 U/mL penicillin, 50 μg/mL streptomycin (Gibco/Invitrogen, Carlsbad, CA, USA) and incubated at 37 °C in a 5% CO2 incubator.

#### 2.2.2. BFPSCs

The fat was manipulated as reported elsewhere [21]. The subcutaneous adipose tissue harvested from the buccal fat pad was enzymatically digested by 0.075% type I collagenase (30 min at 37 °C). The stromal vascular fraction rich in BFPSCs was isolated by centrifugation at 1200 g for 10 min, then plated in Dulbecco’s modified Eagle’s medium (DMEM; Sigma-Aldrich, Milan, Italy), and supplemented with 10% fetal bovine serum (FBS), 50 U/mL penicillin, 50 μg/mL streptomycin, and 2 mM L-glutamine (Sigma-Aldrich).

### 2.3. Success Rate of Isolation of DPSCs and BFPSCs

According to the protocol described by Nakajima et al. [22], successful isolation was defined when the cell culture reached the third passage (P3) without any contamination. Cells were observed daily under an optical microscope until the day when cell adhesion was observed, and colonies were formed.

### 2.4. Cell Number and Morphology Evaluation

The number of viable cells was quantified through manual count. Briefly, 50 μL of the sample was mixed with 50 μL of 0.4% trypan blue by gently pipetting, and then 10 μL of the mix was loaded into each chamber of a Bürker chamber. Counts were performed in triplicate by using a 10X objective. As described elsewhere [23], cells were seeded at a concentration of 5000 cells/well in a 24-well plate, for 1 day. After fixing in 4% paraformaldehyde in phosphate buffer saline (PBS), cells were stained with Alexa488-Phalloidin and diamidino-2-phenylindole (DAPI, Life Technologies, Milan, Italy) to mark the actin network and nuclei, respectively. Images were acquired with a Nikon Eclipse Ti-E microscope using a Nikon Plan 20×/0,10 (Nikon Instruments, Amsterdam, Netherlands) [24]. Quantitative morphometric characterization was accomplished using MORPHEUS, a Fiji/ImageJ2 plugin designed for the unbiased and reproducible analysis of cell morphometry from images acquired by fluorescence microscopy [25]. First, MORPHEUS segmented and automatically recognized most of the isolated cells present in each input image. Then, the algorithm evaluated the morphometry of the selected cells by means of 12 different shape descriptors (i.e., area, perimeter, best fitting ellipse (BFE) major axis, BFE minor axis, BFE aspect ratio, BFE angle, circularity, roundness, solidity, Feret’s diameter, Feret’s angle, and minimum caliper diameter) allowing for a multivariate statistical approach.

### 2.5. Proliferation Assay

To evaluate their proliferation, the BFPSCs and DPSCs were seeded at a density of 1000 cells/well in 96-well culture dishes, and viability was assessed by CellTiter-Glo^®^ (Promega, Milan, Italy) according to the manufacturer’s protocol at 24, 48, and 72 h. The CellTiter-Glo^®^ Luminescent Cell Viability Assay is a homogeneous method for determining the number of viable cells in a culture based on quantitation of adenosine triphosphate (ATP) as a marker of metabolically active cells. The amount of ATP is directly proportional to the number of viable cells in culture, and for this reason this assay can be used as an indicator of cell proliferation (as reported by the manufacturer).

### 2.6. Osteodifferentiation Assays

To assess osteogenic differentiation, cells were cultured in osteogenic medium (OM), constituted by alpha-modified Eagle’s medium supplemented with 50 μg/mL ascorbic acid, 10–8 M dexamethasone, and 10 mM beta-glycerophosphate (Sigma-Aldrich). After 14 days of culture, the expression of alkaline phosphatase (ALP) was evaluated through IHC staining (ALP kit, supplied by Sigma-Aldrich). The formation of mineralized nodules was assessed by Von Kossa staining, after 8 weeks of culture.

### 2.7. Flow Cytometry Analysis of DPSCs and BFPSCs Phenotypes

The expression of typical MSCs markers was analyzed by flow cytometry. In detail, cells were identified as CD105-, CD44-, CD73- and CD90-positive, or CD45-, and CD3-negative.

Standard labeling protocol was performed with the following fluorochrome-conjugated antibodies and isotypic controls: human CD105 PE (Invitrogen, Camarillo, CA, USA), CD73 FITC (kindly provided by Prof. Malavasi, University of Turin), CD44 FITC, CD45 PerCP, CD3 APC, IgG1 PE and IgG2a PerCP (Miltenyi Biotech, Bergisch Gladbach, Germany), and FITC-conjugated IgG1 (Immunostep). As a further control, unstained cells were also examined. About 105 events/sample were used for capture with CellQuest software. Data were analyzed with FlowLogic software (Miltenyi Biotech).

### 2.8. Osteocalcin Evaluation

Following the manufacturer’s protocol, Osteocalcin (OCN) was measured in the growth medium (GM) and osteogenic medium (OM) media by means of an Osteocalcin Elisa kit (KAQ1381 Invitrogen Corporation, Camarillo, CA, USA), after 21 days of culture.

### 2.9. RNA Extraction and Real-Time PCR Analysis

Total RNA was extracted using a PureLink RNA Mini Kit (Ambion, Life Technologies Italy, Milan, Italy). For the quantitative real-time polymerase chain reaction (qRT-PCR), 1 μg total RNA was transcribed into complementary DNA by MultiScribe^®^ Reverse Transcriptase (High-Capacity cDNA Reverse Transcription Kit, Thermo Fisher Scientific, Waltham, MA, USA), and the PCR analysis was then assessed using TaqMan probes from Roche. Transcript abundance, normalized to 18 s mRNA expression, was expressed as a fold increase over a calibrator sample. The qRT-PCR was performed on a 7900HT Fast Real-Time PCR System (Applied Biosystems, Life Technologies Italy, Milan, Italy). Specific primers and probes were designed using the Universal Probe Library—Assay Design Center Roche Life Science software.e (www.lifescience.roche.com, last accessed date: 12/28/2021).

### 2.10. Statistical Analysis

The Mann–Whitney U test was performed to compare the age at extraction and the period from the plated day until the day of the confirmation of cells between the success and failure groups; *p* < 0.05 and *p* < 0.01 were considered statistically significant. Hotelling’s *T*^2^ test was used as a multivariate hypothesis test in the quantitative morphometric analysis (BFPSC vs. DPSC comparison).

## 3. Results

### 3.1. Success Rates in Isolating MSCs from BFP and DP

**Figure 1:**
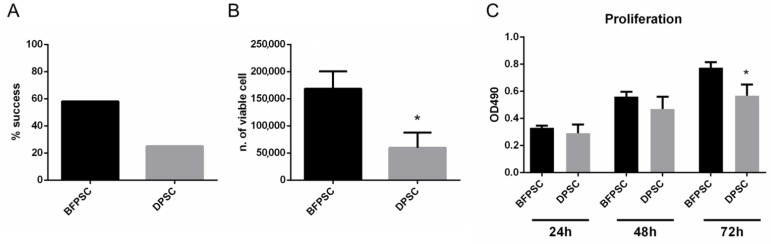
Mesenchymal stem cells (MSC) isolation from buccal fat pad stem cells (BFPSCs) and dental pulp stem cells (DPSCs). (**A**) the success rate in isolating stem cells was higher for BFPSCs than for DPSCs. (**B**) The number of viable cells was significantly reduced in cultures of DPSCs than of BFPSCs. (**C**) The proliferation of BFPSCs and DPSCs was reported at 12, 48, and 72 h of culture. A significant difference was evident at 72 h The symbol (*) indicates a significant difference between BFPSC and DPSC, considering a *p*-value < 0.05.

Five days after culture, both the BFPSCs and DPSCs adhered to the cell culture dishes, and their isolation was evident from an optical microscope observation of cell adhesion and formation of colonies. Cell adhesion was not observed in cases where stem cells were not isolated, even 10 days after seeding. Cells were cultured in vitro for 3 passages, and the isolation of BFPSCs and DPSCs was successful in 7 out of 12 (58%) and 3 out of 12 (25%) of the retrieved samples, respectively. As reported in Figure 1A, the success rate in isolating stem cells was higher for BFP than for DP.

The growth of BFPSCs and DPSCs was assessed at the first passage, showing a significantly reduced number of viable cells in cultures of DP compared to BFP, as shown in Figure 1B. The proliferation of BFPSCs and DPSCs was comparable in the first 48 h of culture, whereas the results were significantly lower for DPSCs than for BFPSCs after 72 h of culture (Figure 1C). These data suggest that BFP is a valuable source from which to derive MSCs.

### 3.2. BFPSCs Show a MSC Immunophenotype

**Figure 2:**
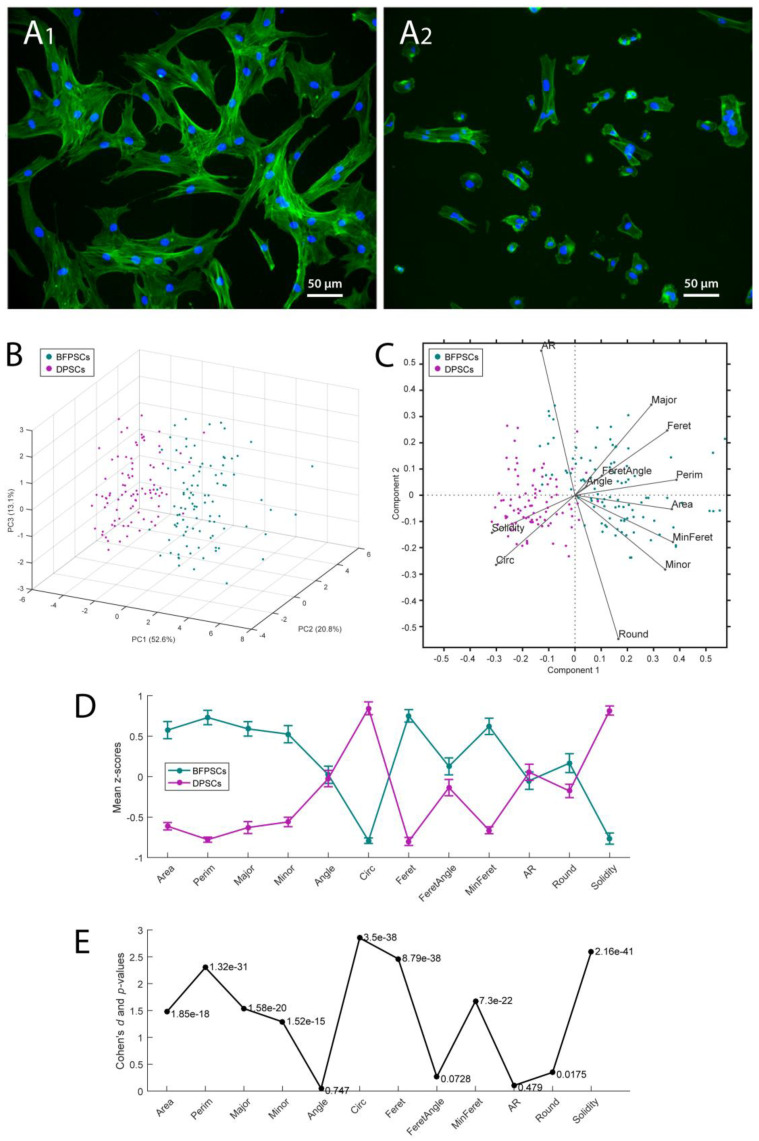
Morphological characterization of BFPSCs (**A1**) and DPSCs (**A2**). Staining with Alexa488-Phalloidin and DAPI showing the actin network and nuclei, respectively, of BFPSCs and DPSCs. Magnification 200×. (**B**) Two sets of cells consisting of *n* = 96 BFPSCs (from 24 independent pictures) and *n* = 90 DPSCs (from 15 independent pictures) were used for quantitative morphometry assessment. Twelve shape descriptors were computed for each cell and principal component (PC) analysis allowed representing the two experimental groups in the reduced space of the first three PCs (together accounting for more than 86% of the total dataset variability). (**C**) Biplot representing the two groups of cells in the space of the first two PCs together with the projections of the 12 original axes (one for each shape descriptor considered). (**D**) For a comparative evaluation of the most influential features, mean ± standard error of measurement (SEm) was evaluated for the two groups separately, starting with the z-scores of each descriptor (i.e., after distribution standardization). (**E**) For each single shape descriptor, the related effect size (Cohen’s *d*) and the *p*-value resulting from the marginal univariate *t*-test are reported.

BFPSCs (Figure 2A1) showed the typical spindle-shaped morphology of MSCs, while DPSCs (Figure 2A2) showed a more polygonal shape. An automated morphometric analysis allowed for a quantitative evaluation of such a difference. Specifically, 12 shape descriptors were evaluated over *n* = 96 BFPSCs and *n* = 90 DPSCs, and the resulting data were subjected to principal component (PC) analysis for dimensionality reduction. As expected, the two groups of cells were clearly separated when represented in the space of the first two or three PCs (Figure 2B,C). Accordingly, a multivariate Hotelling’s *T*^2^ test comparing the two cell types across all descriptors returned an extremely low *p*-value (<10^−50^) confirming the sharp separation. The marginal analysis of the individual descriptors (Figure 2D,E) showed that size and edge smoothness represented the most significant differences between the two experimental groups. In particular, the BFPSCs exhibited areas, perimeters, and Feret diameters significantly larger than the DPSCs did, while circularity and solidity values were significantly larger within the DPSC pool.

**Figure 3:**
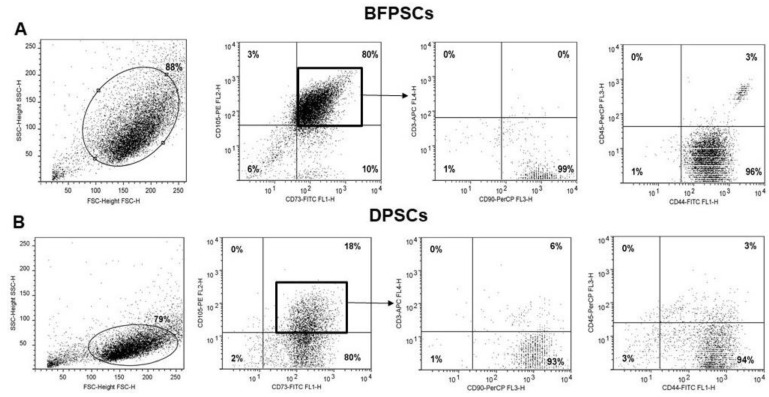
Cytofluorimetric characterization of BFPSCs and DPSCs. (**A**) In in vitro cultures, BFPSCs developed a population of about 80% of the MSCs expressing CD105, CD73, CD90 and CD44 and were basically negative for CD3 and CD45, whereas (**B**) DPSCs cultures were 18% of the MSCs. The squares indicate double positive cells for CD73 and CD105. As indicated by the arrow, the double positive CD105/CD73 cells also expressed CD90 and CD44.

A flow cytometry analysis confirmed the different morphology of BFPSCs and DPSCs by examination of their physical parameters (forward- and side-scatter) (Figure 3). Both BFPSCs and DPSCs expressed markers of MSC phenotypes, such as CD105, CD44, CD73, and CD90, while lacking expression of CD45 (a marker of hematopoietic cells) according to the requirements proposed by the International Society for Cellular Therapy (ISCT) for defining MSCs [26]. The percentages of expression of these markers were not different between BFPSCs and DPSCs and are reported in Table 2.

About 80% of the BFPSCs expressed the markers CD105, CD44, CD73, and CD90, while being negative for CD45 and CD3 (Figure 3A). In the DPSC cultures, the cell population result was more heterogeneous, and only 18% of cells expressed MSCs markers (Figure 3B). These data can explain the different MSC yields from these sources, which thus may influence stem cell retrieval.

### 3.3. BFPSCs and DPSCs Differentiate into Osteoblast-Like

**Figure 4:**
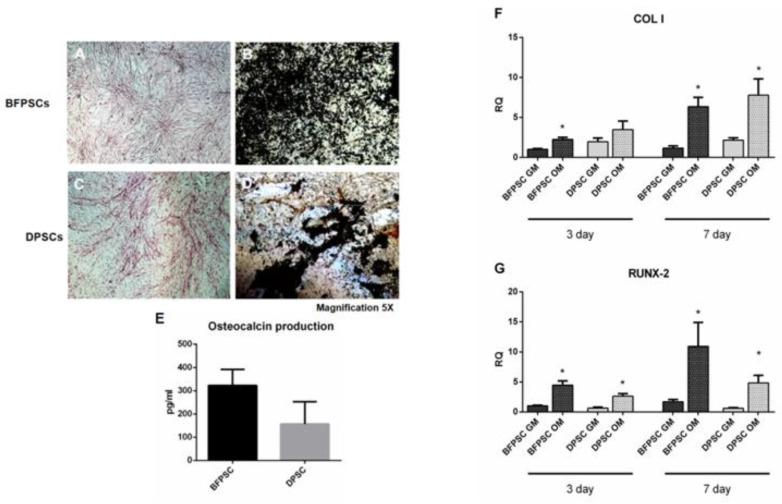
Osteodifferentiation capability. Both BFPSCs and DPSCs differentiate into osteoblast-like cells expressing alkaline phosphatase (ALP) (**A**,**C**), and were able to deposit mineralizing granules (**B**,**D**). The BFPSCs and DPSCs also released osteocalcin in the culture media. Magnification 5× (**E**) osteocalcin detection performed through ELISA 21 days after osteoinduction. (**F**,**G**). qRT-PCR analysis of early osteogenic markers: collagen type I (F), RUNX-2 (G), performed on BFPSCs and DPSCs under basal conditions (GM) and in differentiating medium (OM) at 3 and 7 days. The symbol (*) indicates a significant difference between GM and OM conditions, considering a *p*-value < 0.05.

We investigated the osteogenic differentiation potential of the isolated BFPSCs and DPSCs. After 14 days of culture in the osteogenic medium, both the BFPSCs and the DPSCs were able to differentiate into osteoblast-like cells, as shown by the expression of ALP (Figure 4A,C) and by the mineralization granules released in the culture (Figure 4B,D). We also dosed the release of osteocalcin in the cell culture media without significant differences showing between the BFPSCs and the DPSCs, even though the level of osteocalcin was higher in the BFPSCs than in the DPSCs (Figure 4E). To assess the early osteogenic differentiation of BFPSCs and DPSCs, the transcript levels of the collagen type I (Figure 4F) and RUNX-2 (Figure 4G) genes were evaluated through quantitative RT-PCR at 3 and 7 days. As it is possible to appreciate from Figure 4, the BFPSCs and DPSCs were properly induced toward the osteogenic commitment, and the bone markers were constantly higher in the osteodifferentiated cells than in the control.

## 4. Discussion

Among the SCs described in the literature for regenerative purposes, human adult SCs represent a promising tool bereft of ethical issues and with extremely limited safety concerns [21]. Although bone marrow-derived stem cells (BMSCs) have traditionally been the prototypical MSCs, their highly invasive harvest procedure has prompted research toward easily attainable alternatives endowed with similar multilineage potential, such as ASCs. Nonetheless, SCs derived from different anatomical sites could be more prone to respond to stimuli naturally secreted in the body area of origin, and thus for the treatment of dental defects, SCs isolated from oral cavity should be more effective than ASCs.

In the oral cavity, there are different sources of SCs, such as DPSCs [3], SHED [14,15], PDLSCs [16] and BFPSCs [18,19]. All have been proposed as promising candidates for tissue engineering protocols in the dental and maxillofacial fields [27]. Strangely, though, studies of SCs deriving from more than one site owing to the same individual are not easily reported in the scientific literature. This prevents direct intra-individual comparison of the phenotypical features and effective differentiative abilities of different types of SCs. Therefore, to eliminate this possible lack of knowledge, we collected and compared BFPSCs to DPSCs isolated from the same donor, in a series of twelve patients.

Interestingly, the isolation success rate differed greatly between BFPSCs (58%) and DPSCs (25%). Only a few previous studies dealt with the success rate of the isolation of different types of MSCs. For instance, Biebak et al. [28] optimized the protocols to increase the isolation yield of umbilical cord blood MSCs (UCB-MSCs) up to 63%, while BMSCs were used as a positive control (success rate 100%). Nakajima et al. [22] investigated the success rate of isolating DPSCs and SHED, reporting 70% and 82%, respectively. Neither of these studies harvested the two cell types from the same donor, most probably owing to the apparent difficulty in collecting cells from two different sites within the same patient, and hence derives the relevance of the protocol proposed in the present work. As remarked before, BFPSCs were chosen as representative ASCs following the study by Broccaioli and colleagues [21], who pointed out their similarity to traditional ASCs.

After harvesting, BFPSCs were more numerous in terms of cells alive and proliferated more than DPSCs at 72 h. Both the BFPSCs and the DPSCs expressed typical MSCs markers, such as CD105, CD44, CD73, and CD90, but the yield of cells expressing these markers was higher in the BFPSC population than it was in the DPSCs. DPSCs and BFPSCs show a different morphology, and we suggest that it depends on their different origin of isolation. Previously, we published data concerning the different morphologies and expression patterns of cytokines and growth factors between ASCs and SHEDs [15]. Even though all these cells are MSCs, they derive from specific tissues. Retaining some memory of those tissues, they thus exhibit some tissue-specific properties in addition to more generic multipotential properties [29,30]. As for the osteodifferentiation capability of the two cell types, both promoted the formation of mineralized tissue in vitro, although the level of expression of osteocalcin was higher in the BFPSCs. This is consistent with the accumulating evidence supporting the usage of DPSCs [31,32,33,34,35] and ASCs [36,37,38,39,40] as promising tools for bone regeneration.

With the increasing number of studies involving different intraoral cell sources, however, more extensive research will be needed to attain safe and predictable regenerative medicine procedures. The efficiency of an isolation protocol varies according to several parameters and may be conveniently tailored to ex vivo manipulation [41]. For instance, the Good Manufacturing Process requires serum-free medium (SFM) or auto serum to reduce the risk of viral and prion contamination. Moreover, long-term MSC cultures may undergo malignant transformation [42], making it mandatory to obtain the highest number of cells in the shortest time possible. Both DPSCs [43] and ASCs [44] could be expanded successfully in culture media without animal-derived antigens (xenofree).

Within the limits of this study, the data showed that BFP is a viable intraoral source of MSCs for regenerating bone defects. Here, BFPSCs could properly grow and differentiate, and seemed even to outperform DPSCs. Under this perspective, our results may become relevant soon when a choice is to be made whether to employ extracted teeth or adipose tissue for bone regenerative protocols.

## Figures and Tables

**Table 1:** Gender, age and site of the grafts.

Patient	Gender	Age	Tooth Site	Buccal Fat Pad (BFP) Site
1	M	20	18	R
2	F	26	28	L
3	F	23	28	L
4	F	27	28	L
5	M	22	18	R
6	M	30	18	R
7	F	21	28	L
8	M	27	18	R
9	M	26	28	L
10	M	24	18	R
11	F	25	18	R
12	M	22	28	L

**Table 2:** The values indicated represent the mean ±SD percentage of expression of these MSC markers by BFPSCs and DPSCs.

	CD44	CD73	CD90	CD105
**BFPSCs**	95.4 ± 2.1	84.5 ± 7.8	98.9 ± 0.8	76.8 ± 23
**DPSCs**	97.9 ± 1.2	95.1 ± 3.4	98.9 ± 0.7	65.3 ± 30.1

## Data Availability

The datasets generated during and/or analysed during the current study are available from the corresponding author on reasonable request.

## References

[1] Genova T., Roato I., Carossa M., Motta C., Cavagnetto D., Mussano F. (2020). Advances on Bone Substitutes through 3D Bioprinting. Int. J. Mol. Sci..

[2] Liao H.-T., Chen C.-T. (2014). Osteogenic potential: Comparison between bone marrow and adipose-derived mesenchymal stem cells. World J. Stem Cells.

[3] Gronthos S., Mankani M., Brahim J., Robey P.G., Shi S. (2000). Postnatal human dental pulp stem cells (DPSCs) in vitro and in vivo. Proc. Natl. Acad. Sci. USA.

[4] Bourin P., Bunnell B.A., Casteilla L., Dominici M., Katz A.J., March K.L., Redl H., Rubin J.P., Yoshimura K., Gimble J.M. (2013). Stromal cells from the adipose tissue-derived stromal vascular fraction and culture expanded adipose tissue-derived stromal/stem cells: A joint statement of the International Federation for Adipose Therapeutics and Science (IFATS) and the International Society for Cellular Therapy (ISCT). Cytotherapy.

[5] Zuk P.A., Zhu M., Mizuno H., Huang J., Futrell J.W., Katz A.J., Benhaim P., Lorenz H.P., Hedrick M.H. (2001). Multilineage cells from human adipose tissue: Implications for cell-based therapies. Tissue Eng..

[6] Zuk P.A., Zhu M., Ashjian P., De Ugarte D.A., Huang J.I., Mizuno H., Alfonso Z.C., Fraser J.K., Benhaim P., Hedrick M.H. (2002). Human adipose tissue is a source of multipotent stem cells. Mol. Biol. Cell.

[7] Kokai L.E., Marra K., Rubin J.P. (2014). Adipose stem cells: Biology and clinical applications for tissue repair and regeneration. Transl. Res..

[8] Roato I., Belisario D.C., Compagno M., Verderio L., Sighinolfi A., Mussano F., Genova T., Veneziano F., Pertici G., Perale G. (2018). Adipose-Derived Stromal Vascular Fraction/Xenohybrid Bone Scaffold: An Alternative Source for Bone Regeneration. Stem Cells Int..

[9] Pers Y.-M., Rackwitz L., Ferreira R., Pullig O., Delfour C., Barry F., Sensebe L., Casteilla L., Fleury S., Bourin P. (2016). Adipose Mesenchymal Stromal Cell-Based Therapy for Severe Osteoarthritis of the Knee: A Phase I Dose-Escalation Trial. Stem Cells Transl. Med..

[10] Perdisa F., Gostyńska N., Roffi A., Filardo G., Marcacci M., Kon E. (2015). Adipose-Derived Mesenchymal Stem Cells for the Treatment of Articular Cartilage: A Systematic Review on Preclinical and Clinical Evidence. Stem Cells Int..

[11] Roato I., Belisario D.C., Compagno M., Lena A., Bistolfi A., Maccari L., Mussano F., Genova T., Godio L., Perale G. (2019). Concentrated adipose tissue infusion for the treatment of knee osteoarthritis: Clinical and histological observations. Int. Orthop..

[12] Kunimatsu R., Nakajima K., Awada T., Tsuka Y., Abe T., Ando K., Hiraki T., Kimura A., Tanimoto K. (2018). Comparative characterization of stem cells from human exfoliated deciduous teeth, dental pulp, and bone marrow-derived mesenchymal stem cells. Biochem. Biophys. Res. Commun..

[13] Nakajima K., Kunimatsu R., Ando K., Ando T., Hayashi Y., Kihara T., Hiraki T., Tsuka Y., Abe T., Kaku M. (2018). Comparison of the bone regeneration ability between stem cells from human exfoliated deciduous teeth, human dental pulp stem cells and human bone marrow mesenchymal stem cells. Biochem. Biophys. Res. Commun..

[14] Miura M., Gronthos S., Zhao M., Lu B., Fisher L.W., Robey P.G., Shi S. (2003). SHED: Stem cells from human exfoliated deciduous teeth. Proc. Natl. Acad. Sci. USA.

[15] Mussano F., Genova T., Petrillo S., Roato I., Ferracini R., Munaron L. (2018). Osteogenic Differentiation Modulates the Cytokine, Chemokine, and Growth Factor Profile of ASCs and SHED. Int. J. Mol. Sci..

[16] Seo B.-M., Miura M., Gronthos S., Bartold P.M., Batouli S., Brahim J., Young M., Robey P.G., Wang C.-Y., Shi S. (2004). Investigation of multipotent postnatal stem cells from human periodontal ligament. Lancet.

[17] Martin D.E., De Almeida J.F.A., Henry M.A., Khaing Z.Z., Schmidt C.E., Teixeira F.B., Diogenes A. (2014). Concentration-dependent effect of sodium hypochlorite on stem cells of apical papilla survival and differentiation. J. Endod..

[18] Meshram M., Anchlia S., Shah H., Vyas S., Dhuvad J., Sagarka L. (2019). Buccal Fat Pad-Derived Stem Cells for Repair of Maxillofacial Bony Defects. J. Maxillofac. Oral Surg..

[19] Khojasteh A., Hosseinpour S., Rezai Rad M., Alikhasi M., Zadeh H.H. (2019). Buccal fat pad-derived stem cells with anorganic bovine bone mineral scaffold for augmentation of atrophic posterior mandible: An exploratory prospective clinical study. Clin. Implant Dent. Relat. Res..

[20] Mussano F., Ferrocino I., Gavrilova N., Genova T., Dell’Acqua A., Cocolin L., Carossa S. (2018). Apical periodontitis: Preliminary assessment of microbiota by 16S rRNA high throughput amplicon target sequencing. BMC Oral Health.

[21] Broccaioli E., Niada S., Rasperini G., Ferreira L.M., Arrigoni E., Yenagi V., Brini A.T. (2013). Mesenchymal Stem Cells from Bichat’s Fat Pad: In Vitro Comparison with Adipose-Derived Stem Cells from Subcutaneous Tissue. Biores. Open Access.

[22] Nakajima K., Kunimatsu R., Ando K., Hiraki T., Rikitake K., Tsuka Y., Abe T., Tanimoto K. (2019). Success rates in isolating mesenchymal stem cells from permanent and deciduous teeth. Sci. Rep..

[23] Genova T., Petrillo S., Zicola E., Roato I., Ferracini R., Tolosano E., Altruda F., Carossa S., Mussano F., Munaron L. (2019). The Crosstalk Between Osteodifferentiating Stem Cells and Endothelial Cells Promotes Angiogenesis and Bone Formation. Front. Physiol..

[24] Canullo L., Genova T., Mandracci P., Mussano F., Abundo R., Fiorellini J.P. (2017). Morphometric Changes Induced by Cold Argon Plasma Treatment on Osteoblasts Grown on Different Dental Implant Surfaces. Int. J. Periodontics Restor. Dent..

[25] Ruffinatti F.A., Genova T., Mussano F., Munaron L. (2020). MORPHEUS: An automated tool for unbiased and reproducible cell morphometry. J. Cell. Physiol..

[26] Dominici M., Le Blanc K., Mueller I., Slaper-Cortenbach I., Marini F., Krause D., Deans R., Keating A., Prockop D., Horwitz E. (2006). Minimal criteria for defining multipotent mesenchymal stromal cells. The International Society for Cellular Therapy position statement. Cytotherapy.

[27] Tassi S.A., Sergio N.Z., Misawa M.Y.O., Villar C.C. (2017). Efficacy of stem cells on periodontal regeneration: Systematic review of pre-clinical studies. J. Periodontal Res..

[28] Bieback K., Kern S., Klüter H., Eichler H. (2004). Critical parameters for the isolation of mesenchymal stem cells from umbilical cord blood. Stem Cells.

[29] Hynes K., Menicanin D., Mrozik K., Gronthos S., Bartold P.M. (2014). Generation of functional mesenchymal stem cells from different induced pluripotent stem cell lines. Stem Cells Dev..

[30] Demarco F.F., Casagrande L., Zhang Z., Dong Z., Tarquinio S.B., Zeitlin B.D., Shi S., Smith A.J., Nör J.E. (2010). Effects of morphogen and scaffold porogen on the differentiation of dental pulp stem cells. J. Endod..

[31] De Mendonça Costa A., Bueno D.F., Martins M.T., Kerkis I., Kerkis A., Fanganiello R.D., Cerruti H., Alonso N., Passos-Bueno M.R. (2008). Reconstruction of large cranial defects in nonimmunosuppressed experimental design with human dental pulp stem cells. J. Craniofac. Surg..

[32] Jahanbin A., Rashed R., Alamdari D.H., Koohestanian N., Ezzati A., Kazemian M., Saghafi S., Raisolsadat M.A. (2016). Success of Maxillary Alveolar Defect Repair in Rats Using Osteoblast-Differentiated Human Deciduous Dental Pulp Stem Cells. J. Oral Maxillofac. Surg. Off. J. Am. Assoc. Oral Maxillofac. Surg..

[33] Petridis X., Diamanti E., Trigas G.C., Kalyvas D., Kitraki E. (2015). Bone regeneration in critical-size calvarial defects using human dental pulp cells in an extracellular matrix-based scaffold. J. Cranio-Maxillo-Facial Surg. Off. Publ. Eur. Assoc. Cranio-Maxillo-Facial Surg..

[34] Daltoé F.P., Mendonça P.P., Mantesso A., Deboni M.C.Z. (2014). Can SHED or DPSCs be used to repair/regenerate non-dental tissues? A systematic review of in vivo studies. Braz. Oral Res..

[35] Ercal P., Pekozer G.G. (2020). A Current Overview of Scaffold-Based Bone Regeneration Strategies with Dental Stem Cells. Adv. Exp. Med. Biol..

[36] Corsetti A., Bahuschewskyj C., Ponzoni D., Langie R., Santos L.A.D., Camassola M., Nardi N.B., Puricelli E. (2017). Repair of bone defects using adipose-derived stem cells combined with alpha-tricalcium phosphate and gelatin sponge scaffolds in a rat model. J. Appl. Oral Sci..

[37] Luby A.O., Ranganathan K., Lynn J.V., Nelson N.S., Donneys A., Buchman S.R. (2019). Stem Cells for Bone Regeneration: Current State and Future Directions. J. Craniofac. Surg..

[38] Lin C.-Y., Chang Y.-H., Li K.-C., Lu C.-H., Sung L.-Y., Yeh C.-L., Lin K.-J., Huang S.-F., Yen T.-C., Hu Y.-C. (2013). The use of ASCs engineered to express BMP2 or TGF-β3 within scaffold constructs to promote calvarial bone repair. Biomaterials.

[39] Senarath-Yapa K., McArdle A., Renda A., Longaker M.T., Quarto N. (2014). Adipose-derived stem cells: A review of signaling networks governing cell fate and regenerative potential in the context of craniofacial and long bone skeletal repair. Int. J. Mol. Sci..

[40] Zhao X., Liang M., Li X., Qiu X., Cui L. (2018). Identification of key genes and pathways associated with osteogenic differentiation of adipose stem cells. J. Cell. Physiol..

[41] Tevlin R., McArdle A., Brett E., Chung M.T., Paik K., Seo E.Y., Walmsley G.G., Duldulao C.R., Atashroo D., Zielins E. (2016). A Novel Method of Human Adipose-Derived Stem Cell Isolation with Resultant Increased Cell Yield. Plast. Reconstr. Surg..

[42] Røsland G.V., Svendsen A., Torsvik A., Sobala E., McCormack E., Immervoll H., Mysliwietz J., Tonn J.-C., Goldbrunner R., Lønning P.E. (2009). Long-term cultures of bone marrow-derived human mesenchymal stem cells frequently undergo spontaneous malignant transformation. Cancer Res..

[43] Xiao J., Yang D., Li Q., Tian W., Guo W. (2018). The establishment of a chemically defined serum-free culture system for human dental pulp stem cells. Stem Cell Res. Ther..

[44] Czapla J., Matuszczak S., Kulik K., Wiśniewska E., Pilny E., Jarosz-Biej M., Smolarczyk R., Sirek T., Zembala M.O., Zembala M. (2019). The effect of culture media on large-scale expansion and characteristic of adipose tissue-derived mesenchymal stromal cells. Stem Cell Res. Ther..

